# Multi-function oxidases are responsible for the synergistic interactions occurring between repellents and insecticides in mosquitoes

**DOI:** 10.1186/1756-3305-2-17

**Published:** 2009-04-16

**Authors:** Julien Bonnet, Cédric Pennetier, Stéphane Duchon, Bruno Lapied, Vincent Corbel

**Affiliations:** 1 Laboratoire de Lutte contre les Insectes Nuisibles, Institut de Recherche pour le Développement, 911 Avenue Agropolis, BP 64 501, 34394 Montpellier, France; 2 Centre de Recherches Entomologiques de Cotonou (CREC), Institut de Recherche pour le Développement (IRD), 01 BP 4414 RP, Cotonou, Benin; 3 Laboratoire RCIM UPRES EA 2647/USC INRA 2023, IFR 149 QUASAV, 49045 Angers, cedex, France; 4 University of Greenwich at Medway, Central Avenue, Chatham, Kent ME4 4TB, England, UK; 5 School of Life Science, University of Sussex, Brighton, BN1 9QG, England, UK

## Abstract

**Background:**

With the spread of pyrethroid resistance in mosquitoes, the combination of an insecticide (carbamate or organophosphate) with a repellent (DEET) is considered as a promising alternative strategy for the treatment of mosquito nets and other relevant materials. The efficacy of these mixtures comes from the fact that they reproduce pyrethroid features and that positive interactions occur between insecticides and repellent. To better understand the mechanisms involved and assess the impact of detoxifying enzymes (oxidases and esterases) in these interactions, bioassays were carried out in the laboratory against the main dengue vector *Aedes aegypti*.

**Methods:**

Topical applications of DEET and propoxur (carbamate), used alone or as a mixture, were carried out on female mosquitoes, using inhibitors of the two main detoxification pathways in the insect. PBO, an inhibitor of multi-function oxidases, and DEF, an inhibitor of esterases, were applied one hour prior to the main treatment.

**Results:**

Results showed that synergism between DEET and propoxur disappeared in the presence of PBO but not with DEF. This suggests that oxidases, contrary to esterases, play a key role in the interactions occurring between DEET and cholinesterase inhibitors in mosquitoes.

**Conclusion:**

These findings are of great interest for the implementation of "combination nets" in the field. They support the need to combine insecticide with repellent to overcome insecticide resistance in mosquitoes of public health importance.

## Background

Pyrethroids are currently the only insecticides recommended by the World Health Organization for the treatment of insecticidal materials against mosquitoes of public health importance [1]. The great success of pyrethroids is related to their strong efficacy at low dose, fast killing effect and relative low cost of production. Their low toxicity to humans and stability over time ensure a safe and effective personal protection against a wide range of pests and vectors [2]. Since the 1980s, they have been widely used for house spraying and impregnation of mosquito nets for malaria control [3].

Unfortunately, pyrethroid resistance is now widespread in mosquitoes. Mechanisms of resistance involve target site modification due to mutation within structural receptor genes and metabolic resistance *via *increased detoxification of insecticides [4]. Resistance represents a serious obstacle for vector control as demonstrated recently with insecticide-treated mosquito nets and indoor residual sprayings in Benin [5], as well as control of *Aedes aegypti *during space spraying in the Caribbean [6].

In this context, new molecules and strategies are urgently needed to preserve the efficacy of insecticide-treated materials used in public health [7]. Among the different strategies proposed, the combination of a repellent with a carbamate or an organophosphate (OP) on treated materials showed promising results for malaria vector control under simulated field conditions [8,9]. The strong killing effect of cholinesterase inhibitors added to the high personal protection of repellents reproduced pyrethroid features against several mosquito vectors. For example, a mixture of DEET (the gold standard for synthetic repellent) and propoxur (carbamate) showed equivalent toxicity to deltamethrin at the dose that killed 100% (LD_100_) of susceptible *Ae. Aegypti*. Moreover, on the *kdr *homozygous strain of the same species, the mixture performed significantly better than deltamethrin [10]. This strong efficacy was attributed to the synergistic interactions occurring between propoxur and DEET. These interactions were also observed between an organophosphate, pyrimiphos-methyl and the two repellents DEET and picaridin^® ^on bed nets against *Anopheles gambiae *in both laboratory and field experiments, confirming that this strategy may be promising for the control of pyrethroid resistant mosquitoes [8,9].

However, the physiological mechanisms involved in these interactions remain unclear. While carbamates and OPs inhibit acetylcholinesterase in insects [11], controversies remain over DEET mode of action [12,13] and toxicity in insects [14-16]. Recent studies showed that DEET toxicity may occur through a general perturbation of insect neuronal transmission [17,18].

As previously described by Corbel et al. [19] with pyrethroid-carbamate combinations, synergistic interactions between molecules having different modes of action may result from a general physiological disruption, involving different target sites in the central nervous system. Another hypothesis is the involvement of detoxification enzymes. Indeed, one component of the mixture may interfere with the detoxification of the other, thereby increasing the toxicity of both [20,21]. Such involvement of esterases [22] or oxidases [23] has already been shown in synergism between pyrethroids and OPs. The OP prevents the degradation of the pyrethroid insecticide by competing as enzyme substrates.

In the present study, we investigated through toxicological bioassays (topical applications) the mechanisms involved in DEET and propoxur interactions by using two enzyme inhibitors (PBO and DEF) against the dengue and yellow fever vector, *Aedes aegypti*.

## Methods

### Mosquitoes

The susceptible strain, Bora, of *Ae. aegypti*, originating from French Polynesia, was used for this study. This strain has been colonized in the laboratory for many years and is free of any detectable resistance mechanisms.

### Insecticide, repellent and enzyme inhibitors

Bioassays were carried out with technical grades of active ingredients diluted in acetone. Propoxur (2-isopropoxyphenylmethylcarbamate) 99.6% was provided by Bayer CropScience (Monheim, Germany). DEET (N,N-diethyl-m-toluamide) 97% was provided by Sigma-Aldrich (Saint Quentin Fallavier, France). Enzyme inhibitors, piperonyl butoxide (5-((2-(2-butoxyethoxy)ethoxy)methyl)-6-propyl-1,3-benzodioxole) 90% and S,S,S-tributyl phosphorotrithioate 98.1% were purchased from Fluka (Buchs, Switzerland) and Chem Service (West Chester, PA, USA). Piperonyl butoxide (PBO) is a well known inhibitor of cytochrome-P450 monooxygenases (multi-function oxidases), widely used as a synergist for insecticide treatments [24]. S,S,S-tributyl phosphorotrithioate (DEF) is a specific inhibitor of esterases.

### Topical applications

Topical applications were used to measure the interactions occurring between technical insecticide and repellent on *Ae. aegypti*. This method allows estimating the intrinsic toxicity of a product excluding all other effects linked to mosquito's behaviour, especially when exposed to an irritating or repellent compound. For each product alone or in a mixture, five to eight doses were tested to provide a range of mortality from nil to 100%. Non-blood-fed females of *Ae. aegypti*, aged 2–5 days, were first anaesthetised by extended contact with carbon dioxide and deposited on a chilled plate (4°C) maintaining anaesthesia during manipulation. Fifty females were used for each dose tested. A volume of 0.1 μl of acetone solution (containing the product(s) at the required concentration(s)) was applied on the upper part of female's pronotum using a micro-capillary tube. Fifty females that received 0.1 μl of pure acetone served as control. Enzyme inhibitors were applied at the maximal sub-lethal dose (1000 ng/female for PBO and 500 ng/female for DEF) 1 hour before the main treatment using the same protocol. Females were preserved at 4°C on the chilled plate during this interval of time, to ensure the diffusion of enzyme inhibitor through mosquito body prior to insecticide treatment. After manipulation, females were transferred into plastic cups, provided with sugar solution and held for 24 hours at 27°C and 80% RH. Mortality rates were recorded 24 hours after testing and corrected according to the formula of Abbott [25] in the case of a control mortality > 5%. Data were expressed in nanograms of active ingredient per milligram of mosquito body weight. Three replicates were done for each test using different batches and generations of mosquitoes.

### Analysis of interactions

Dose-effect regression lines of each product (insecticide and repellent) and their mixture were drawn using Probit software [26]. Data were analyzed according to the median-effect method of Chou and Talalay [27] using CalcuSyn software [28]. This software provides an estimation of the median-effect doses (Dm analogous to LD_50_) with their 95% confidence intervals for each product and mixture. The median-effect equation states that:



where *Fa *and *Fu *are the fractions of mosquitoes affected and unaffected respectively by the dose *Dx *of insecticide(s). *m *represents the slope of the regression line and *Dm *the dose required to produce the median effect.

Insecticide, repellent and their mixture were evaluated in three conditions, i.e. first in absence of enzyme inhibitors, then following a pre-treatment of PBO or DEF. Three different binary mixtures of propoxur and DEET were prepared for each experimental situation (without synergist/with PBO/with DEF). The ratio of the mixture was determined by the ratio of the median effect doses of propoxur and DEET obtained in the situation considered. At the ratio chosen, both compounds are expected to make an equal contribution in killing mosquitoes.

The isobologram method of Chou and Talalay [27] was used to assess whether the interactions occurring between DEET and propoxur were synergistic, antagonistic or simply additive in the presence or absence of synergists (PBO and DEF). In this study, the use of isobologram was done as follows: diagonal lines connect doses of propoxur on the x-axis to doses of DEET on the y-axis where each product is in theory equally efficient at killing mosquitoes when applied alone (e.g. isoboles ED_50_, ED_75 _and ED_90 _correspond to a dosage causing 50%, 75% and 90% mortality, respectively). The points relative to each line indicate how much of each product is required to achieve the same effect when applied in a mixture at the ratio chosen. Points below relative line indicate synergism, points close to relative line indicate additive effect, and points above relative line indicate antagonism between the compounds.

## Results

The dose-effect regression lines for DEET, propoxur and their mixtures were fitted by straight lines (goodness of fit, P > 0.05). The highest mortality value in control batches was 13%. The median-effect doses (Dm) for these relationships are listed in Table 1.

**Table 1:** Median-lethal doses of propoxur, DEET and their mixture on susceptible *Ae. aegypti*.

Chemicals	Dm without synergists	Dm with PBO	Dm with DEF
propoxur	1.97 (1.78–2.19)	0.27 (0.17–0.42)	1.01 (0.83–1.24)
DEET	1189 (1088–1299)	611 (596–629)	1078 (1005–1155)
mixture	319 (296–343)	406 (385–428)	128 (119–136)
mixture ratio P:D	1:600	1:2000	1:1000
Slope of the regression lines (± s.e.)			
propoxur	2.83 (0.19)	2.16 (0.18)	1.95 (0.22)
DEET	4.16 (0.42)	3.98 (0.12)	3.18 (0.20)
mixture	3.32 (0.18)	3.76 (0.20)	2.96 (0.14)

Data are expressed in ng of active ingredient per mg of mosquito body weight (± 95% confidence intervals).

**Figure 1:**
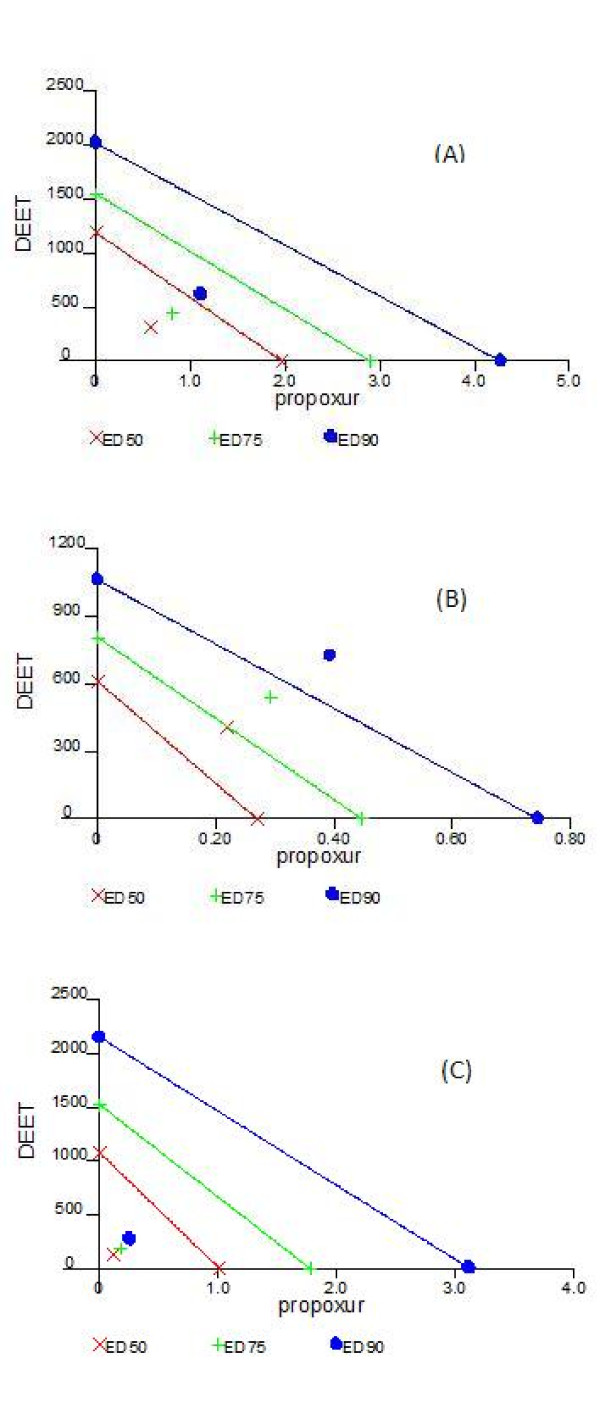
**Isobolograms of insecticidal activity for propoxur, DEET and their mixtures on *Ae. aegypti***. In absence of synergist (A), with PBO (B) and with DEF (C). Diagonal lines connect doses of equipotent activity for each product applied alone. Relative symbols indicate the amount of each product required to induce the same effect in a mixture (at the ratio based on median-effect doses of each product). Points below relative line indicate synergism, points close to relative line indicate additive effect, and points above relative line indicate antagonism between the compounds.

The median-lethal doses on *Ae. aegypti *were 1.97 (1.78–2.19) ng a.i. per mg female for propoxur and 1 189 (1088–1299) ng a.i. per mg female for DEET (Table 1). This result shows the toxicity at high dose of DEET against mosquitoes. According to these values, a binary mixture of propoxur and DEET was prepared at the ratio 1:600. The mixture was more potent at killing mosquitoes than one would expect in the case of a simple additive effect (Figure 1A). This observation confirmed the occurrence of synergistic interactions between DEET and propoxur, regardless the dosage considered (ED_50_, ED_75 _and ED_90_).

In the presence of PBO (1000 ng/female applied 1 hour before the treatment), the toxicity of propoxur and DEET significantly increased (Table 1). The median-lethal doses of propoxur [0.27 (0.17–0.42)] and DEET [611 (596–629)] were about 7 and 2 times lower than that obtained without synergists. This indicates that cytochrome-P450 monooxygenases played a role in the detoxification of these two compounds. According to the values of median-lethal doses obtained with PBO, a second mixture of propoxur and DEET was prepared (ratio 1:2000). In presence of PBO, the mixture did not show any synergistic interactions in *Ae. aegypti *(Figure 1B). Conversely, slight antagonism was observed, the mixture being less efficacious at killing mosquitoes than expected in the case of a simple additive effect (Figure 1B).

Applying DEF at 500 ng/female 1 hour prior to treatment with propoxur and DEET had little effect on toxicity of both compounds (Table 1). The median-lethal dose of propoxur [1.01 (0.83–1.24)] was only 2 times lower than that observed without synergist whereas toxicity of DEET [1078 (1005–1155)] was unchanged. According to the values of median-lethal doses obtained with DEF, a third mixture of propoxur and DEET was prepared (ratio 1:1000). In presence of DEF, the synergistic interactions between the compounds were maintained regardless of the dosage considered (Figure 1C).

## Discussion

In the present study, topical applications of insecticide(s) were carried out on *Ae. aegypti *to better understand the physiological mechanisms involved in synergism between DEET and propoxur.

First, the Isobologram method of Chou and Talalay [27] confirmed the previous observations of Pennetier et al. [10] who demonstrated that the insecticidal activity of DEET and propoxur was enhanced when they are applied together. Our findings also showed that oxidases are able to metabolize the carbamate propoxur but also the repellent DEET, thus confirming previous works of Constantino et al. [29,30]. Conversely, esterases had little impact on toxicity of the two molecules. This can be explained by the absence of an ester bond in DEET which prevents any hydrolytic metabolism by esterases [30].

Most interestingly, pre-treatment with PBO suppressed the synergism previously observed between DEET and propoxur. According to these data, we suggest that cytochrome-P450 monooxygenases are responsible for the enhanced toxicity observed between the repellent and the carbamate. We assume that the competition between DEET and propoxur for the multi-function oxidases increased the toxicity of the mixture. Electrophysiological experiments are now in progress to identify the physiological events underlying DEET and carbamate toxicity in the insect central nervous system [31].

Cytochrome-P450 monooxygenases have been identified as being involved in strong pyrethroid resistance because they are over-expressed in wild resistant mosquitoes [32-34]. Our results suggest that the activity of these enzymes is essential for the synergistic interactions between DEET and propoxur. Now, we can consider whether the overexpression of monooxygenases will enhance the toxicity of repellent and insecticide mixtures against mosquitoes. If yes, this suggests that these mixtures should be an effective tool to manage pyrethroid-resistance based on overexpression of multi-function oxidases. The next step should be to study the interactions between carbamates/OPs and repellents on mosquitoes in which oxidases are overexpressed.

## Conclusion

As shown by Pennetier et al [10], the association of non-pyrethroid insecticide and repellent exhibited pyrethroid features, especially a fast killing effect and excito-repellency properties. The existence of positive interactions between the compounds is a major argument in favour of their possible use in public health. Reduced amounts of active ingredient would provide an effective protection for the treatment of mosquito nets and other relevant materials. The fact that multi-function oxidases are involved in these interactions (and not insecticide-target sites) is of great interest for the implementation of "combination nets" in the field and the management of insecticide resistance in mosquitoes.

In areas where resistance to pyrethroids can no longer be controlled, the use of carbamate (or organophosphate) combined to repellents appears as an effective alternative to pyrethroids, as they show efficacy equivalent to these insecticides in simulated field situations [8,9]. In other situations, such combinations might also be used as a supplement to pyrethroids to retard the spread of resistance. Further investigations in live situations are certainly necessary prior to the use of insecticide and repellent combinations for vector control. The efficacy of these mixtures against mosquitoes has to be assessed in the field on a significant period of time, as well as its cost and safety to human. However, we think that they offer significant advantages for public health intervention. First, as no pyrethroid insecticides are used, their efficacy would not be altered by *kdr *mutation widespread in mosquito populations [35-37]. Using such combinations may also be of great interest in areas where mosquito populations show resistance based on oxidase metabolism [32-34]. As observed in the laboratory, metabolic-based resistance may facilitate synergism between carbamate and repellent when using "two in one" treated materials. In this perspective, these mixtures should be evaluated in areas where mosquitoes show a high and broad range of metabolic-based resistance.

## Competing interests

The authors declare that they have no competing interests.

## Authors' contributions

JB carried out the bioassay studies and drafted most of the manuscript. CP and BL helped to draft the manuscript. SD participated to the bioassay studies. VC conceived the study and helped to draft the manuscript. All authors read and approved the final manuscript.
